# Clearing the outer mitochondrial membrane from harmful proteins via lipid droplets

**DOI:** 10.1038/cddiscovery.2017.16

**Published:** 2017-03-20

**Authors:** Johannes Bischof, Manuel Salzmann, Maria Karolin Streubel, Jiri Hasek, Florian Geltinger, Jutta Duschl, Nikolaus Bresgen, Peter Briza, Danusa Haskova, Renata Lejskova, Mentor Sopjani, Klaus Richter, Mark Rinnerthaler

**Affiliations:** 1 Department of Cell Biology and Physiology, Division of Genetics, University of Salzburg, Salzburg, Austria; 2 Institute of Physiology, Centre of Physiology and Pharmacology, Medical University of Vienna, Vienna, Austria; 3 Laboratory of Cell Reproduction, Institute of Microbiology of AS CR, v.v.i., Prague, Czech Republic; 4 Department of Molecular Biology, University of Salzburg, Salzburg, Austria; 5 Laboratory of Cell Reproduction, Institute of Microbiology of AS CR, v.v.i., Prague, Czech Republic; 6 Faculty of Medicine, University of Prishtina, Prishtinë, Kosova

## Abstract

In recent years it turned out that there is not only extensive communication between the nucleus and mitochondria but also between mitochondria and lipid droplets (LDs) as well. We were able to demonstrate that a number of proteins shuttle between LDs and mitochondria and it depends on the metabolic state of the cell on which organelle these proteins are predominantly localized. Responsible for the localization of the particular proteins is a protein domain consisting of two *α*-helices, which we termed V-domain according to the predicted structure. So far we have detected this domain in the following proteins: mammalian BAX, BCL-XL, TCTP and yeast Mmi1p and Erg6p. According to our experiments there are two functions of this domain: (1) shuttling of proteins to mitochondria in times of stress and apoptosis; (2) clearing the outer mitochondrial membrane from pro- as well as anti-apoptotic proteins by moving them to LDs after the stress ceases. In this way the LDs are used by the cell to modulate stress response.

## Introduction

Mitochondria fulfill a pleiotropy of functions in the cell and are one of the most versatile organelles. Via the electron transport chain and OXPHOS they produce most of the ATP a cell needs. They are involved in fatty acid and amino acid metabolism.^1^ Furthermore they are a major production site of reactive oxygen species (ROS), which can be used as signaling molecules. ROS are, for example, part of an communication network between mitochondria and the nucleus in homeostasis and stress (reviewed in ref. 2). Although a certain level of ROS can be beneficial for the cell,^3^ excessive levels have detrimental effects. It has been known for decades that mitochondria and their dysfunction are heavily involved in the aging process as well as age-associated diseases.^4,5^ Superoxide originating from complexes I and III has the potential to damage mtDNA, proteins and lipids.^6^ During the aging process, ROS levels increase tremendously, resulting in accumulated mitochondrial damage and creates a vicious cycle.^4^ As a result the mitochondrial morphology changes from a tubular network to an isolated, blob-shaped appearence.^6,7^ This change in morphology may represent a way for the cell to separate damaged from undamaged mitochondria.

Systems for counteracting ROS damage include dismutation of superoxide by superoxide dismutases,^8^ refolding of unfolded proteins by chaperones^9^ and the degradation of misfolded proteins by the mitochondrial proteases.^10^ If, however, the accumulated damage exceeds a certain threshold the cell commits to apoptosis leading to specific events: (1) translocation of specific proteins to mitochondria; (2) mitochondrial fission and cristae disorganization; (3) outer membrane permeabilization; (4) calcium influx; (5) release of mitochondrial pro-apoptotic factors such as cytochrome c; and (6) a loss of the mitochondrial membrane potential.^11–13^ Of crucial importance for apoptosis is the shuttling of pro- and anti-apoptotic proteins to mitochondria.^14,15^ Prominent examples are MCL1, BMF, BAX, BCL2, BCL-XL, NOXA, Puma, BAD, BAK, BID, BIK and BIM.^16,17^

Among these BAX is a key protein. After activation it changes its structure, inserts into the outer mitochondrial membrane, and forms a dimer leading to the formation of a higher oligomerized structure. This oligomerization leads to a permeabilization of the outer mitochondrial membrane.^18,19^ In contrast, BCL-XL is a prominent example for proteins that inhibit this oligomerization.^20,21^ The cell is able to remove damaged, non-functional mitochondria by mitophagy.^22^ According to the present article, this is the only mechanism that enables the cell to deal with damaged mitochondria without committing to apoptosis.

In this article, we propose the existence of an alternative mechanism that enables the cell to regulate the translocation of pro- and anti-apoptotic proteins to mitochondria and the initiation of the apoptotic process. We also characterized a special domain in these proteins, responsible for the localization of various proteins to mitochondria as well as their shuttling between mitochondria and lipid droplets (LDs).

## Results

### Identification of the V-domain

**Figure 1:**
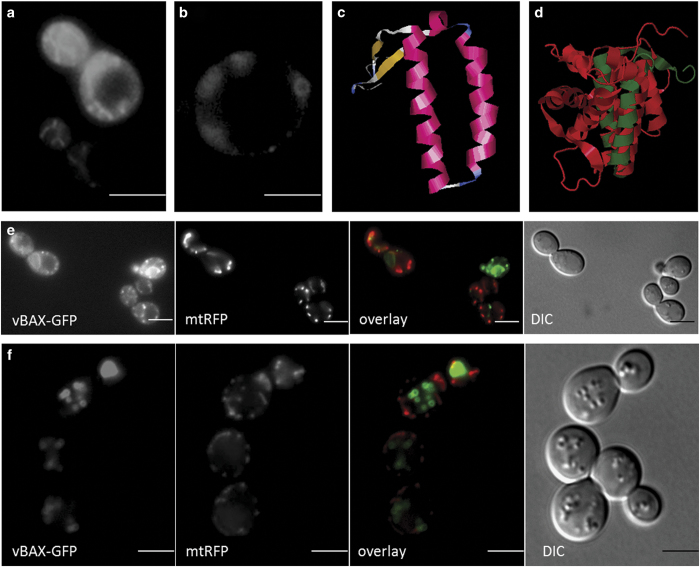
Localization of V-domain proteins *in S. cerevisiae*. (**a**) Localization of the V-domain obtained from Mmi1p in exponentially growing cells. (**b**) Localization of the Mmi1p V-domain in stationary cells. (**c**) Structure prediction of the Mmi1p V-domain (visualized with RasMol). (**d**) Superimposition of the BAX protein (red) with the Mmi1p V-domain (DaliLite v. 3). (**e**) Co-localization of the BAX V-domain fused to GFP with mitochondria (visualized with mtRFP). In exponentially growing cells (harboring a pXY142-mtRFP and a pUG35-vBAX-GFP plasmid) there is a perfect match between the V-domain of BAX and mitochondria (as seen in the overlay section). (**f**) In stationary cells, the V-domain of BAX detaches from mitochondria and localizes to vesicle like structures (as seen in the overlay section). Scale bars, 5 *μ*m.

Besides some of the more well-known pro- and anti-apoptotic proteins there are others, whose involvement in apoptosis is more complex. One of these proteins is TCTP (or its yeast homolog Mmi1p), a highly conserved protein assumed to participate in a wide variety of cellular processes such as apoptosis, stress response, microtubule organization and ion homeostasis.^23,24^ This protein roughly resembles chaperones.^25^ A flexible loop region and a helical domain are the structural characteristics of TCTP.^26^ The flexible loop region harbors a calcium-binding motif,^27^ whereas it is assumed that the helical domain is responsible for tubulin binding.^28^ Besides its many intracellular functions, TCTP is also secreted and can be detected in the extracellular space.^29^ A re-import of this protein is made possible by a special protein-transduction domain that penetrates all biological membranes with high efficiency.^30^ In a previous work, we were able to show that stress is able to initiate a shift of Mmi1p from the cytosol to the outer mitochondrial membrane.^31^ This finding was later on confirmed in mice.^32^ In 2013, we could demonstrate that the binding of Mmi1p to mitochondria is mediated by an *α*-helical domain (V-domain) consisting of two helices.^33^ This was shown by cloning the V-domain of MMI1 via PCR into the yeast vector pUG35 and expressing it as a C-terminal GFP fusion protein. Surprisingly, this domain was found in vesicular structures after yeast cells depleted all nutrients and the culture reached stationary phase (Figure 1a). In a follow-up approach, we tried to identify other proteins containing a V-domain. The structure of the Mmi1p V-domain was predicted with Swiss-Model (http://swissmodel.expasy.org) (Figure 1b) and was then submitted to the ‘DaliLite v. 3’ server (http://ekhidna.biocenter.helsinki.fi/dali_server/start). Among others, BAX, the master regulator of apoptosis, showed a V-domain too. BAX contains nine *α*-helices (reviewed in ref. 34) and *α*5 and *α*6, two amphipathic helices that together form a hairpin show an overlap with Mmi1p (Figure 1c). It was demonstrated that mutations in this two helices prevent mitochondrial localization of BAX.^35,36^ Therefore, we cloned the GFP-tagged V-domain of BAX into the yeast expression vector pUG35. During the early exponential phase, when nutrients are plenty, a mitochondrial localization of this BAX domain was observed (Figure 1e). In mid-log phase, when the concentrations of nutrients drop considerably, yeast cells begin to store energy in form of lipids. Interestingly, during this phase, the V-domain starts to detach from mitochondria and accumulates in vesicular structures (Figure 1f). These vesicles become more prominent during cell growth and reach a maximum in size during stationary phase.

### LD and mitochondrial localization of the V-domain

**Figure 2:**
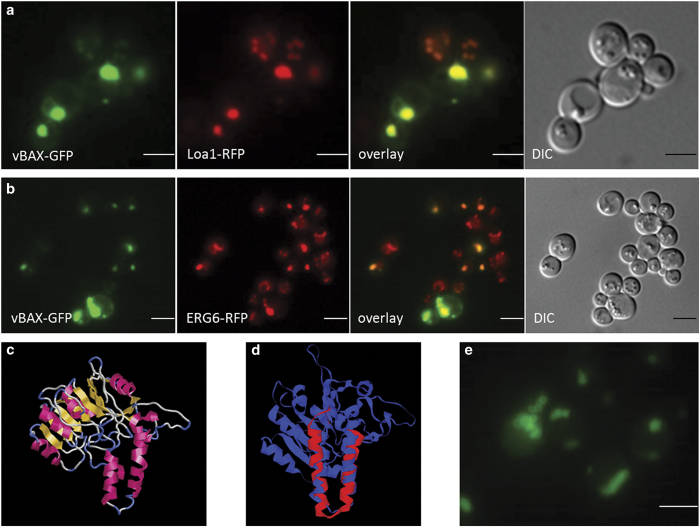
Localization of V-domain proteins at LDs. (**a**) Yeast cells expressing both the GFP-tagged V-domain of BAX (pUG35-V-BAX-GFP) as well as the RFP-tagged LD marker protein Loa1 (LOA1-kanMX-RFP). (**b**) Yeast cells expressing both the GFP-tagged V-domain of BAX (pUG35-V-BAX-GFP) as well as the RFP-tagged LD marker protein ERG6 (ERG6-kanMX-RFP). In both cases, a perfect overlap between the V-domain and LDs was observed (see overlays). (**c**) A structure prediction of Erg6 (predicted with Swissmodel; displayed with RasWin). (**d**) An overlay between the V-domain of BAX and Erg6p (displayed with DaliLite v. 3). (**e**) Localization of the V-domain of Erg6 in yeast cells. Scale bars, 5 *μ*m*.*

By co-localizing the V-domain of BAX with a RFP-tagged Erg6p (Delta(24)-sterol C-methyltransferase) and a RFP-tagged Loa1p (lysophosphatidic acid acyltransferase), we demonstrated that these vesicles are in fact LDs (Figures 2a and b). Of special interest is Erg6p, because this protein localizes to LDs,^37^ as well as to the outer mitochondrial membrane.^38^ For Erg6p, a potential 3D structure based on homology modeling (http://swissmodel.expasy.org/) was predicted (Figures 2c and d) as no NMR or X-ray defraction structure data are available. A domain resembling the V-domain was GFP-tagged by cloning it into pUG35. This V-domain peptide is localized either to mitochondria (early log phase) or to LDs (late exponential and stationary phase) in yeast cells. Therefore, we hypothesize that we have identified a domain responsible for communication between mitochondria and LDs. Compared to Mmi1p the V-domain of BAX and Erg6p seems to have a higher affinity for LDs than for the outer mitochondrial membrane.

**Figure 3:**
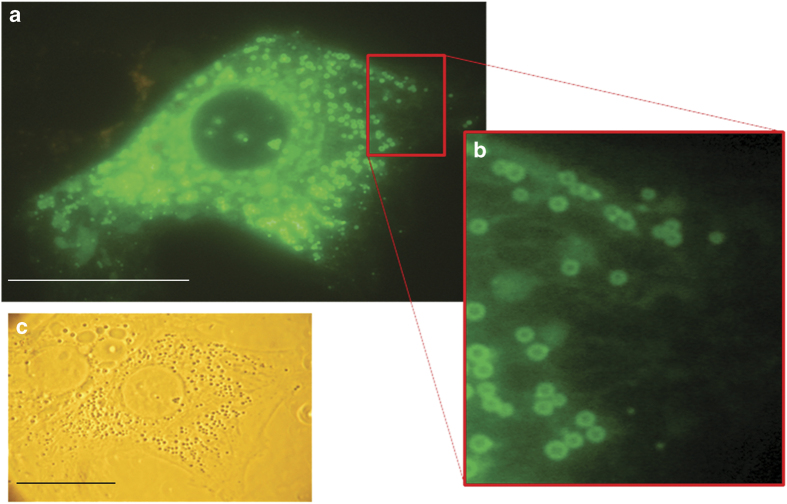
Localization of V-domain proteins in primary rat hepatocytes. (**a**) Primary rat hepatocyte expressing the GFP-tagged V-domain of BAX after transfection with the vector pEGFP-N3-V-BAX. (**b**) Magnification of **a** showing vesicular as well as tubular structures. (**c**) DIC of the primary rat hepatocyte. Scale bars, 10 *μ*m*.*

**Figure 4:**
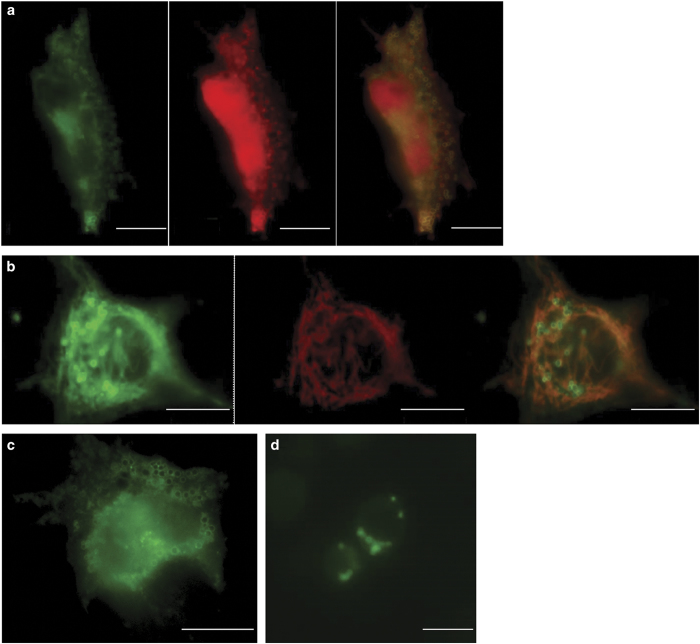
V-domain proteins localize to LDs and to mitochondria. (**a**) HepG2 cells transfected with the bi-cistronic vector pIRES2-V-domain (BAX)/PLIN2-RFP show a partial co-localization between the RFP and GFP signal confirming the localization of the V-domain to LDs. The GFP-signal (V-domain of BAX), the RFP signal (Plin2) and the overlay (yellow) is shown. (**b**) HepG2 cells transfected with the bi-cistronic vector pIRES2-V-domain (BAX)/TOMM20-RFP, show a partial co-localization between the RFP and GFP signal confirming the mitochondrial localization of the V-domain. The GFP-signal (V-domain of BAX), the RFP signal (TOMM20) and the overlay (yellow) is shown. White bar, 10 *μ*m. (**c**, **d**) The LD localization of the V-domain of BCL-XL in HepG2(**c**) and yeast cells (**d**) is shown. Scale bars, 10 *μ*m (**a**–**c**); 5 *μ*m (**d**).

The localization of this Mito-LD-shuttle domain was also tested in mammalian cells. Preferred cells for these studies were hepatocytes, because they have a very high content of LDs. In an initial step the V-domain of murine, BAX was cloned into the mammalian shuttle vector pEGFP-N3 and transfected into primary rat hepatocytes. Fluorescence microscopic visualization revealed both a tubular network and vesicular structures (Figure 3). To prove mitochondrial as well as LD localization, the bi-cistronic vector pIRES2 was used for the following constructs: pIRES2-V-domain (BAX)/TOMM20-RFP, pIRES2-V-domain (BAX)/PLIN2-RFP and pIRES2-V-domain (BAX)/PLIN3-RFP. In higher eukaryotes, different types of LDs can be distinguished: smaller, more peripheral LDs and larger, more centrally localized LDs. Plin3 is a marker protein for peripheral LDs, while Plin2 is a marker protein for larger ones.^39,40^ TOMM20 is a translocase of the outer mitochondrial membrane.^41^ Transfection of the human liver cancer cell line HepG2 revealed a co-localization of the V-domain from the murine BAX protein with TOMM20-RFP (Figure 4b) and PLIN2-RFP (Figure 4a) and only a partial co-localization with PLIN3-RFP (Supplementary Figure 1). This experiment demonstrates the evolutionary conserved function of the V-domain as a Mito-LD-shuttle domain. This indicates that the V-domain can only attach to large LDs. According to DaliLite, BCL-XL also harbors a V-domain. A GFP-tagged version showed a clear LD localization in HepG2 cells (Figure 4c) and yeast cells (Figure 4d).

### LD localization of the full-length proteins Mmi1p/TCTP and mBAX

**Figure 5:**
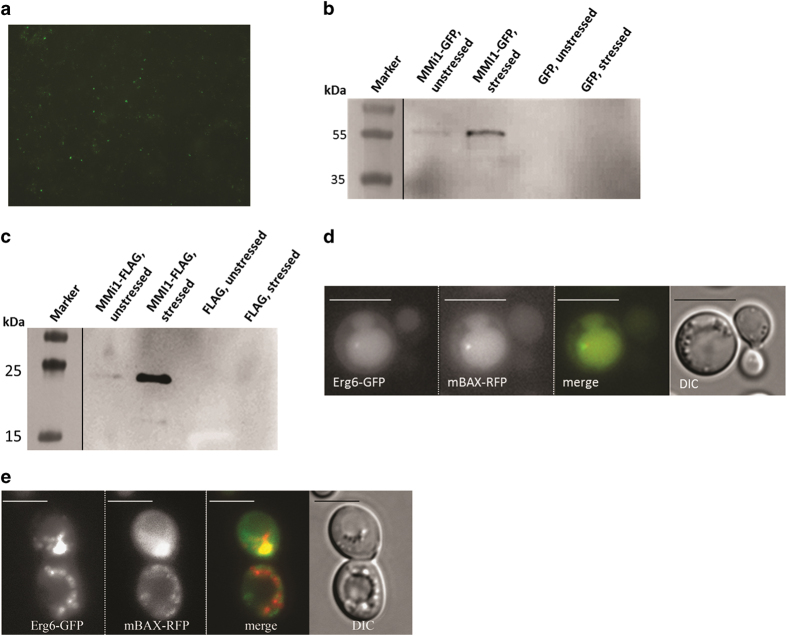
LD localization of full-length proteins. (**a**) Fluorescence (GFP) image of isolated and purified yeast LDs. Mmi1-GFP is clearly attached to purified LDs. (**b**) Immunoblot of purified LDs detecting the Mmi1p-GFP fusion. Heat stress at 46 °C clearly causes the localization of this fusion protein to LDs. (**c**) Immunoblot of purified yeast LDs detecting the Mmi1p-FLAG fusion. Again, 46 °C heat stress clearly leads to a localization of this fusion protein to LDs. (**d**) Co-localization of the LD marker Erg6-GFP with mBAX-RFP in the vacuole of yeast cells. (**e**) Co-localization of the LD marker Erg6-GFP with mBAX-RFP in the cytosol of yeast cells. Scale bar, 5 *μ*m.

To demonstrate that full-length proteins also have the ability to shuttle between mitochondria and LDs, an yeast strain was constructed expressing a GFP-tagged version of full-length Mmi1p. LDs were isolated from stressed (10 min 46 °C) and unstressed yeast cultures. Only LDs from the stressed cultures showed a fluorescence signal (Figure 5a), a fact also confirmed by western blot analysis. In times of stress, a strong band at ~50 kDa (predicted MW for Mmi1-GFP: 47 kDa) appeared, whereas blots from LDs from unstressed cultures revealed only faint signals (Figure 5b). To exclude an influence from the GFP-tag, a FLAG-tagged version of Mmi1p was created and analyzed in the same way. Mmi1p-FLAG (predicted MW: 20.58 kDa) was accumulated in LDs of stressed cells, whereas only low amounts were found in LDs from unstressed cells (Figure 5c). Finally, a mass spectral analysis was performed with LDs from stressed/apoptotic and unstressed yeast cultures. Mmi1p could only be detected in LDs from apoptotic yeast cells.

Working with full-length murine BAX in yeast cells turned out to be difficult, because murine BAX induces apoptosis in yeast.^42,43^ There is no known BAX homolog in yeast cells, although a possible candidate is under investigation.^44^ However, there are also no anti-apoptotic counterparts, resulting in an upregulated cytotoxic effect of BAX. Cloning BAX into the vector p416GPD-RFP and expression of the resulting BAX-RFP-fusion in the *Saccharomyces cerevisiae* strain BY4741, which contains already a pESC-HIS-ERG6-GFP plasmid resulted in very few and slow-growing yeast cells. Co-localization of the full-length BAX with Erg6p was observed, but mobile LDs (Supplementary Video 1) were predominantly found in the vacuole (Figure 5d). In some cases, faint BAX-GFP signals co-localizing with LDs in the cytosol were detected (Figure 5e). This BAX-localization underlines its cytotoxic function and suggests a detoxifying role for LDs in yeast, which led to the following hypothesis: mobile LDs (Supplementary Video 2) attach to mitochondria after stress and V-domain containing proteins are shuttled from mitochondria to LDs. This offers the possibility to reset ‘mitochondrial clock’ after stress ends. Protein-loaded LDs ultimately detach from mitochondria and are degraded in the vacuole. As a positive side effect the degradation of LDs results in a release of neutral lipids. These lipids form a pool for the recovery of membranes and provide energy, a prerequisite for starting growth after a period of stress.

### Role of the V-domain in apoptosis in clearing the OMM from unwanted proteins

**Figure 6:**
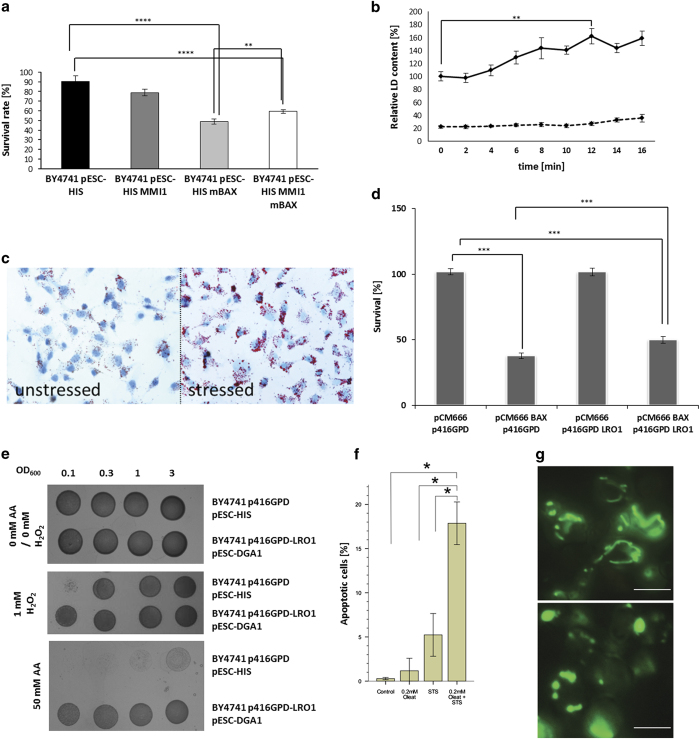
The physiological role of LDs in time of stress. (**a**) The following strains were analyzed: BY4741 pESC-HIS; BY4741 pESC-HIS MMI1; BY4741 pESC-HIS mBAX; and BY4741 pESC-HIS MMI1 mBAX. The shift to the carbon source galactose induced the expression of either Mmi1p, mBAX or the combination of both. Mmi1p overexpression clearly reduced the mBAX-induced apoptotic cell death. The values represent the mean±S.E.M. of 20 biological replicas, the significance of difference was analyzed by Student *t*-test. (**b**) Nile Red staining of LDs in yeast cells after application of 42 °C heat stress. The continuous line represents the wild-type BY4741, the dotted line the quadruple mutant nearly devoid of LDs. The values represent the mean±S.E.M. of five biological replicas, the significance of difference was analyzed by Studentś *t*-test. (**c**) SudanIII stained LDs in HepG2 cells before and after the addition of 50 *μ*M hydrogen peroxide. (**d**) The following strains were analyzed: BY4741 pCM666 p416GPD; BY4741 pCM666-mBAX p416GPD; BY4741 pCM666 p416GPD-LRO1; and BY4741 pCM666-mBAX p416GPD-LRO1. Lro1p is constitutively expressed, whereas the expression of mBAX is induced by the addition of 50 mg/l doxycycline. Stimulation of LDs clearly protects against mBAX-induced apoptotic cell death in yeast cells. The values represent the mean±S.E.M. of 20 biological replicas for the strains BY4741 pCM666 p416GPD and BY4741 pCM666 p416GPD-LRO1 and 82 biological replicas for BY4741 pCM666-mBAX p416GPD and BY4741 pCM666-mBAX p416GPD-LRO1, the significance of difference was analyzed by Studentś *t*-test. (**e**) Yeast cells harboring the two vectors p416GPD and pESC-HIS and cells overexpressing LRO1 (p416GPD-LRO1; constitutive expression) as well as DGA1 (pESC-HIS-DGA1; galactose induced expression) were spotted onto plates containing 50 mM acetic acid or 1 mM hydrogen peroxide. The LD overproducing strains are resistant to hydrogen peroxide and especially acetic acid at a concentration at which the wild-type cannot grow at all. (**f**) HepG2 cells grown in the presence of either 0.2 mM oleate or 0.05 *μ*M sraurosporine or in the combination of both. The resulting increase in LD numbers clearly increases the apoptotic cell death induced by staurosporine. The values represent the mean±S.E.M. of three biological replicas, the significance of difference was analyzed by Studentś *t*-test. (**g**) A yeast strain devoid of LDs (deletion of *are1*, *are2*, *dga1* and *lro1*) and transformed with the plasmid pYX142-mtRFP has clearly fragmented mitochondria. A wild-type (BY4741) carrying the same plasmid shows a stable mitochondrial network. Scale bar, 5 *μ*m.

In mammalian cells, TCTP translocates to mitochondria during apoptosis and interacts with two anti-apoptotic proteins: Mcl-1 and BCL-XL. The result is a decrease of apoptotic cell death (reviewed in ref. 45). The role of the yeast Mmi1p protein at mitochondria is much less clear. Therefore, we tested if this protein has an anti-apoptotic/anti-BAX function. The murine BAX gene and the yeast *MMI1* gene were cloned into the yeast expression vector pESC-HIS, both under control of an inducible, bidirectional GAL1/10 promoter. Addition of galactose leads to the expression of both Mmi1p and mBAX. Expression of mBAX alone causes a clear increase in cell death (survival rate of 49%), whereas a combination leads to a statistically significant increase in cell survival (survival rate 59%, *P*-value=0.0046) (Figure 6a). This indicates that Mmi1p has anti-apoptotic properties too.

To test our hypothesis that LDs have an important role in modulating stress response by clearing the OMM, we checked the amount of LDs after stress induction. According to our theory, the number of LDs should increase. In yeast cells, stress was induced in two different ways. Either by expression of mBAX or by heat shock at 46 °C. LDs were stained with Nile Red, a fluorescent hydrophobic dye that selectively stains neutral lipids.^46^ Response to heat stress is very fast and after only 4 min, an ~10% increase in LD content (Figure 6b) could be observed. LD numbers further increase until a maximum is reached after 12 min (~60% increase; *P*=0.0038). After this point, the number of LDs decreases and starts to rise again after 16 min. A possible explanation for this cyclic appearance/disappearance of LDs could be: after 4 min of stress the content of LDs starts to increase removing potentially harmful proteins from the OMM. The LDs with their protein cargo are then moved to the vacuole for degradation (after 12 min of stress) and a new cycle is started (after 16 min of stress). Besides heat-shock treatment, an increase in LDs was confirmed after expression of mBAX under control of the tetracycline inducible promoter tetO_7_ (yeast transformed with plasmids pCM666 or pCM666-mBAX). Doxycycline addition triggers the expression of mBAX, leading to severe stress and the induction of apoptotic cell death. Sixteen hours after addition of doxycycline a 269.34% (*P*-value<0.0001) increase in LD numbers was observed. These LD ‘waves’ resemble the cyclic release of cytochrome c in mammalian cells.^47^ A correlation between stress and LD numbers was also tested in HepG2 cultures. Cells were treated with 50 *μ*M hydrogen peroxide for 16 h and were stained with Sudan III (ref. 48) after fixation. As can be seen in Figure 6c, the size as well as number of LDs increases significantly. In another approach, HepG2 cells transfected with the vector pEGP-N3-V-domain-BAX-GFP were treated with staurosporine, a kinase inhibitor that induces apoptosis. Confirming our results obtained with hydrogen peroxide, the LD content increases enormously in apoptotic hepatic cells (Supplementary Figure 2).

### Role of LDs in stress response

In follow-up experiments, the role of LDs in stress response was tested. In yeast cells, the number of LDs was boosted with the overexpression of *LRO1*. This gene encodes a phospholipid:diacylglycerol acyltransferase, which forms triacylglycerols out of diacylglycerols. The resulting surplus of triacylglycerols in yeast cells (transformed with the vector p416GPD-*LRO1*) led to an increased number of LDs visualized by a co-expression of the V-domain of mBAX (vector Yeplac181-MET25-V-domain-BAX-GFP) (Supplementary Figure 3). In yeast cells transformed with the plasmid pCM666-mBAX, expression and shuttling of BAX to mitochondria was induced by the addition of doxycycline (50 mg/l). After 16 h, a clear induction of apoptosis was observed and cell survival dropped to 37% (*P*<0.0001). Interestingly, overproduction of Lro1p induced a partial redirection of mBAX to LDs and cell survival increased to 49% (*P*=0.0003) (Figure 6d). We also tested other stressors (hydrogen peroxide and acetic acid) and LD number was further increased by co-expressing Lro1p and Dga1p at the same time. Dga1, a diacylglycerol acyltransferase, also stimulates triacylglycerol synthesis. It turned out that yeast cells (BY4741 p416GPD-LRO1 pESC-DGA1) overexpressing both, Lro1p and Dga1p, were resistant to high concentrations of acetic acid as well as hydrogen peroxide as shown by spot test experiments (Figure 6e). In addition, we created a strain that was deleted for *ARE1*, *ARE2*, *DGA1* and *LRO1* genes at the same time. It has been published that this quadruple mutant is devoid of LDs.^49^ Minor amounts of LDs were still visible (visualized by expressing the GFP-tagged V-domain of mBAX), but their number and size were dramatically decreased compared to the wild-type. This strain showed a reduced resistance to hydrogen peroxide and acetic acid (Supplementary Figure 4). However, this effect is less pronounced than the increased resistance to acetic acid upon LD stimulation. A correlation between LDs and mitochondria could also be demonstrated in another way: we show here that deficiency of LDs in the quadruple mutant results in the progressive fragmentation of the tubular mitochondrial network in yeast cells (Figure 6g). According to our hypothesis, harmful proteins accumulate in the outer mitochondrial membarne in the quadruple mutant causing to the observed phenotype.

**Figure 7:**
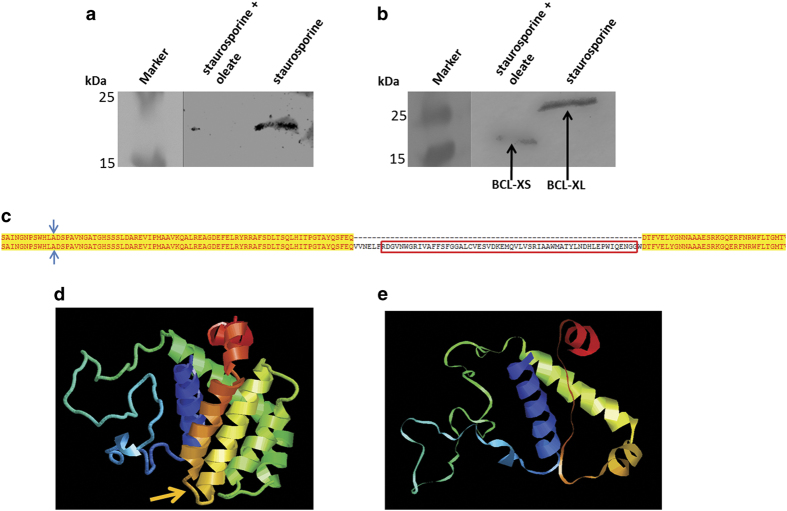
The LD apoptosis paradox. (**a**) Immunoblot of purified human mitochondria detecting the human BAX protein. Staurosporine (0.5 *μ*M, 6 h) induces a shift of BAX to mitochondria, whereas a combination of staurosporine and oleate (0.2 mM) leads to a disappearance of BAX from the outer mitochondrial membrane. (**b**) Immunoblot of purified human mitochondria detecting human BCL-XL/XS. Staurosporine induces a shift of BCL-XL (predicted MW: 26 kDA) to mitochondria, whereas a combination of staurosporine and oleate leads to a redirection of BCL-XL to LDs and at the same time to a translocation of BCL-XS (predicted MW: 18.8 kDa) to mitochondria. (**c**) Sequence alignment of BCL-XL (from amino acid 49–219) and BCL-XS. The difference between these two splice variants is the V-domain marked by the red box. The antibody-binding site is marked by blue arrows. (**d**) Structure prediction of BCL-XL (visualized with RasMol). (**e**) Structure prediction of BCL-XS (visualized with RasMol). The difference between these two isoforms is the V-domain marked by a yellow arrow.

Similar experiments were carried out with HepG2 cells where oleate was added to cell cultures. Oleate has been demonstrated to increase the number of LDs. Apoptosis was induced by addition of 0.05 *μ*M staurosporine. After 24 h, HepG2 cells were fixed, nuclei were stained with DAPI and the morphology of DAPI stained nuclei was used to identify viable, apoptotic and necrotic cells. Addition of staurosporine leads to a clear increase in apoptotic cell death (~5%). The number of apoptotic cells increases more than threefold when combining staurosporine and oleate (~17%; Figure 6f). This result was surprising because an anti-apotpotic effect was expected. Therefore mitochondria were isolated from HepG2 cells that were either treated with 0.05 *μ*M staurosporine or a combination of staurosporine and oleate. Western blot analysis confirmed that addition of staurosporine induced a translocation of BAX and BCL-XL to mitochondria (Figures 7a and b). After addition of oleate both proteins completely disappeared from the outer mitochondrial membrane. In case of oleate-treated cells BCL-XS, a pro-apoptotic splice variant of BCL2L1,^50^ translocated to mitochondria (Figure 7b) and this protein seems to be responsible for the increased cell death in oleate-treated HepG2 cells. Compared with BCL-XL, BCL-XS cannot be removed because the V-domain in this splice variant is missing (Figures 7c–e).

## Discussion

**Figure 8:**
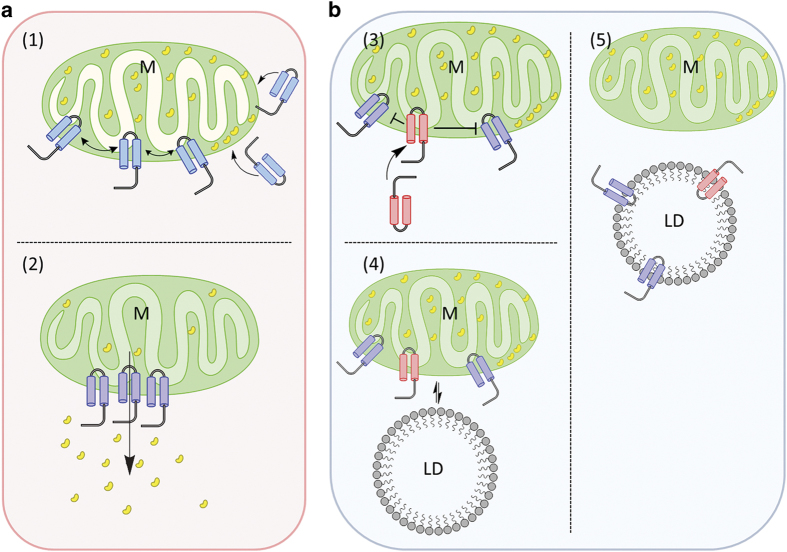
The OMM clearing model via LDs. In **a**, the death pathway is summarized; in **b**, the survival mode is depicted. M indicates mitochondria; LD, lipid droplets. The blue double helical structures indicate the *α*5-*α*6 helices of BAX that are responsible for mitochondrial localization. The red double helical structures indicate the V-domains of Mmi1/TCTP and BCL-XL. The yellow beans represent cytochrome c. In (1), BAX, in response to cellular stress, is translocating from the cytosol to the outer mitochondrial membrane, forming the MAC. In (2), cytochrome c is released that is leading to apoptotic cell death. In (3), BCL-XL as well as TCTP are translocating to mitochondria and both are inhibiting the formation of MAC. In (4), TCTP, BCL-XL and BAX are still present in the outer mitochondrial membrane, although the apoptotic program has stopped. In (5), all pro- and anti-apoptotic proteins are removed from mitochondria by inserting into LDs. Figure has been created by using the Free Trial version of ChemDraw from PerkinElmer (Waltham, MA, USA).

LDs are often named the forgotten organelle. They have been underestimated for quite a while, but finally shifted into the focus of research in the past decade.^51,52^ LDs are originating from the ER^53^ and consist of a neutral lipid core surrounded by a protein-decorated phospholipid monolayer. The hydrophobic core is mainly composed of triacylglycerols (TGs) and cholesterylesters.^40,51^ To preserve their structural integrity, LDs have a unique protein composition, allowing them to interact with other organelles and release or synthesize triacyglycerols.^54^ Besides their birthplace, the ER, LDs are also found in contact with other organelles such as mitochondria and peroxisomes.^55^ Inside the cell LDs are highly mobile and can shuttle between organelles (Supplementary Video 2). The life of a LD ends in the vacuole/lysosome. It was demonstrated that this is actually a type of autophagy termed lipophagy.^56^ LDs are storage organelles for neutral lipids which meet the cell’s requirement for energy, membranes and lipid-derived molecules. There is evidence that LDs also have a role in protein modifications, histone storage and antioxidant defense.^57–59^ The direct interaction with most, if not all organelles enables a direct lipid transfer and could be a tool to prevent lipotoxicity.^60^ Of special interest is the mitochondria-LD-junction mediated via PLIN5.^61^ It is assumed that this interaction enables a flux of fatty acyl-CoAs for oxidation that is essential for energy production. We demonstrate that LDs have an important role in clearing the mitochondrial outer membrane from unwanted proteins and reset the ‘mitochondrial clock’. This is probably a way to rescue mitochondria from being removed via mitophagy. Our hypothesis is briefly summarized in Figure 8: an apoptotic stimulus leads to a conformational change in the pro-apoptotic protein BAX, which re-localizes from the cytosol to mitochondria. After integration into the outer mitochondrial membrane via the V-domain BAX oligomerizes with BAX and BAK and forms a mitochondrial-apoptosis-induced channel (MAC). Formation of MAC marks the point-of-no-return, since it leads to the release of cytochrome c and subsequently to apoptotic cell death. Apoptotic death can be prevented by TCTP or BCL-XL. Both proteins contain a structurally similar V-domain and localize to mitochondria after external stress. TCTP/Mmi1 prevents oligomerization of BAX and formation of MACs and in this way exerts its anti-apoptotic activity. In this work, we show that Mmi1p, similar to human TCTP, can prevent BAX-induced apoptosis. The effect is not as distinct as in mammals, but cell survival is clearly improved from 49 to 59%. TCTP and BAX are not part of healthy mitochondria and have to be removed. LDs are in close contact with mitochondria and TCTP, as well as BAX, can be transferred from mitochondria to LDs via the V-domain. As a direct consequence the number of LDs increases in times of stress. We have shown this for BAX expression, hydrogen peroxide and heat stress in yeast as well as staurosporine and hydrogen peroxide-induced apoptosis in hepatocytes. Our experiments also indicate that BAX decorated LDs are highly toxic and have to be moved to the vacuole for degradation. In the vacuole, LDs are lysed, providing energy for the restart of the cell cycle. Besides Mmi1p/TCTP and BAX we have been able to demonstrate that other proteins, such as BCL-XL and yeast Erg6p, harbor a V-domain. Our data clearly indicate that this domain is a shuttle domain between mitochondria and LDs. In current literature there is growing evidence that LDs are involved in stress response. In murine lymphoma cells, for example, etoposide, an anticancer drug of the topoisomerase inhibitor class, induces an increase in LD numbers.^62^ Emerging data also demonstrate that several tumors utilize LDs for survival. In colon cancer and human breast cancer cells, increased LD numbers were detected.^63,64^ Our results indicate that these tumors use LDs to redirect BAX and other pro-apoptotic proteins from mitochondria to LDs. This way the progression of the apoptotic program is prevented which represents a key event in reaching malignancy.

Taken together LDs are able to collect pro- as well as anti-apoptotic proteins from mitochondria. This either attenuates or increases apoptosis, an effect that obviously depends on the cell type and the metabolic state. In yeast, a higher number of LDs provide the cell with a higher resistance against apoptosis. In HepG2 cells more LDs cause a higher sensitivity towards induction of apoptosis. HepG2 cells redirect both BAX and BCL-XL from mitochondria to LDs, but induce a translocation of BCL-XS to mitochondria that finally kills them. Similar effects have been observed by other groups as oleate induces apoptosis in 3T3-L1 adipocytes^65^ but rescues INS-1E *β*-cells from apoptosis.^66^

## Materials and Methods

### Yeast strains

All strains used in this study are based on the *S. cerevisiae* strain BY4741 (MATa his3Δ1 leu2Δ0 met15Δ0 ura3Δ0) and the null mutants of the same genetic background were obtained from the EUROSCARF deletion collection. The strains were grown at 28 °C in either complex medium (YPD) (1% (w/v) yeast extract, 2% (w/v) peptone and 2% (w/v) d-glucose) or synthetic complete glucose medium (SC-glucose) (2% (w/v) d-glucose, 0.17% (w/v) yeast nitrogen base without amino acids, 0.5% ammonium sulphate and 10 ml of complete dropout mixture (0.2% Arg, 0.1% His, 0.6% Ile, 0.6% Leu, 0.4% Lys, 0.1% Met, 0.6% Phe, 0.5% Thr, 0.4% Trp, 0.1% Ade, 0.4% Ura, 0.5% Tyr) per liter). The quadruple mutant (*Δare1, Δare2, Δdga1, Δlro1*) was created by mating of the appropriate parent strains from the EUROSCARF collection, diploid selection, sporulation and spore dissection after zymolyase digestion using a micromanipulator (Singer, MSM System series 200, Roadwater, UK). All yeast strains used and created for this study are summarized in Supplementary Table 1.

### Cloning experiments

For PCR amplifications the NEB Q5 High-Fidelity DNA Polymerase (NEB, Ipswich, MA, USA) was used. All restriction enzymes were provided by either Promega (Mannheim, Germany), NEB or Thermo Scientific (Waltham, MA, USA). For Gibson Assembly the NEB Gibson Assembly Master Mix was used according to the manufacturer’s protocol. The cloning strategies, sequence of primers (obtained from Sigma-Aldrich, Darmstadt, Germany) enzymes used for cloning and the target vectors are summarized in Supplementary Table 2. All constructs were sequenced by Eurofins-MWG-OPERON (Ebersberg, Germany).

### Chromosomal integration

For chromosomal integration C-terminal RFP fusion proteins were created. Integration cassettes containing RFP as well as kanMX4 were amplified via PCR from the template plasmid pRFP. An aliquot of 1 *μ*g of the PCR product was transformed into yeast cells, chromosomal integration was selected by plating the cells on YPD plates containing 200 *μ*g/ml G418 (Geneticin, Sigma-Aldrich). Integration of the cassette in the correct chromosomal region, creating an RFP fusion, was controlled by PCR.

### Fluorescence microscopy

The distribution of various fusion proteins (or RFP fusions) was analyzed with a ×100 PlanApochromat objective (NA = 1.4) either using an Olympus IX-81 inverted microscope equipped with a Hamamatsu Orca/ER digital camera (Hamamatsu Photonics, Hamamatsu City, Japan) and an Olympus (Tokyo, Japan) CellR detection and analyzing system (GFP filter block U-MGFPHQ, exc. max. 488, em. max. 507; RFP filter block U-MWIY2, exc. max. 545–580, em. max. 610; DAPI filter block U-MNUA2, exc. max. 360–370, em. max. 420–460), a Leica TCS SP5 AOBS confocal laser scanning system (Leica Microsystems, Wetzlar, Germany) coupled to a Leica DMI 6000 Cs inverted microscope, a Carl Zeiss AG Axioscope (Oberkochen, Germany) or Nikon (Tokyo, Japan) Eclipse Ni-U equipped with a DS-Fi2 digital camera.

### ImmunoBlot

Equal amounts of LDs (normalized to OD_600_) were mixed with sample buffer, were loaded to and separated in 15% SDS-PAGE gels and then blotted on protran BA85 nitrocellulose membranes (Schleicher & Schuell BioScience GmbH, Dassel, Germany) (250 mA, 90 min at RT). After blocking the membranes with MTBS-T (25 mM TRIS pH 7.6, 137 mM NaCl, 0.1% TWEEN 20, 5% nonfat milk powder) for 90 min at RT and washing for 30 min with TBS-T the primary-antibodies diluted in MTBS-T were added and incubated overnight at 4 °C under constant shaking. The antibodies were diluted as follows: GFP-antibody (B-2) HRP (sc-9996 HRP; Santa Cruz; 1 : 1000); FLAG M2 antibody (F3165; Sigma-Aldrich; 1 : 500); BAX-antibody (D2E11) (5023; Cell Signal Technologies; 1 : 1000); BCL-XL-antibody (54H6) (2764; Cell Signal Technologies; 1 : 1000). After three washing steps with TBS-T (3×10 min at RT) the membranes were incubated with the secondary-antibody, diluted in MTBS-T (5% w/v milk powder in TBS-T) (Polyclonal rabbit anti-mouse immunoglobulins/HRP; P0161; Dako; 1 : 25000). Detection was carried out with the Pierce (Thermo Fisher Scientific, Waltham, MA, USA) ECL western blotting substrate according the manufacturerś instructions.

### Lipofection of primary liver cells and HepG2

Primary liver cells were grown on glass slides in 24-well plates and lipofected 24 h after perfusion. In case of human HepG2 cells, cells were grown to 80–90% confluency on glass slides. Lipofection took place in six-well plates according to the manufacturer' protocol. Glass slides were then taken out of the 24/6-well plates and were washed with PBS buffer and fixed for 10 min at 37 °C in 4% para-formaldehyd (in PBS). The cells were washed three times with PBS and enclosed in a Mowiol-DABCO solution.

### LD isolation

Starting with an OD_600_ of 0.1 yeast cell were grown for 24 h in YPD medium. After centrifugation, the cells were washed and resuspended in 20 ml of isolaton buffer 1 (0.1 M Tris-HCl, 10 mM DTT pH9.4) and incubated for 15 min at 28 °C. After centrifugation and washing, the cells were resuspended in 25 ml isolation buffer 2 (1.2 M Sorbitol, 20 mM Tripotassium phosphate pH 7.4) supplemented with 0.5 mg/ml zymolyase and incubated for 1 h at 28 °C. Yeast cells were then pelleted and resuspended in 15 ml of a 200 mM Tris pH 7.4 buffer. After cell wall digestion the cells were lysed with 10 strokes in a potter homogenizer and centrifugated for 5 min at 4000×*g*. The supernatant was then centrifuged at 50 000 ×*g* for 10 min. The next centrifugation step was carried out in an ultracentrifuge at 100 000 ×*g* for 30 min. Special self-made suction devices were used to collect LDs floating on top. To cleanse the samples of cytosolic contaminations they were mixed with sucrose and sodium carbonate (end concentrations of 25% and 10 mM, respectively) and layered on top of a 60% sucrose cushion. The sample was then overlaid with 10 mM sodium carbonate followed by the 200 mM Tris pH 7.4 buffer. After ultracentrifugation at 29 000 r.p.m. (100 000×*g*) for 30 min the purified LDs were collected.

### Protein identification in delipidated LDs

LDs were mixed with the same volume of diethyl ether and vortexed thoroughly. After a centrifugation step of 20 000×*g* for 15 min the layer containing the ether was removed and the ether was evaporated. The proteins in the liquid layer were then precipitated using one-tenth of its volume of 100% TCA. Samples were kept on ice for 30 min before centrifugation (30 min at 14 000 r.p.m.). The supernatant was removed and the pellet was washed thoroughly using aceton. Proteins were reduced, alkylated and digested with the ProteoExtract All-in-One Trypsin Digestion Kit (Merck Millipore, Billerica, MA, USA). Peptides were loaded onto the trap column (PepSwift Monolithic Trap Column, Dionex, Thermo Fisher Scientific, Waltham, MA, USA) and desalted with 0.1% (v/v) heptafluorobutyric acid at a flow rate of 10 *μ*l/min. After 5 min, trap and separation column (PepSwift Monolithic Nano Column, 100 *μ*m×25 cm, Dionex) were coupled with a switching valve and the peptides were eluted with an acetonitrile gradient (Solvent A: 0.1% (v/v) FA/0.01% (v/v) TFA/5% (v/v) ACN; solvent B: 0.1% (v/v) FA/0.01% (v/v) TFA/90% (v/v) ACN; 5–45% B in 60 min) at flow rate of 1 *μ*l/min at 55 ° C. The HPLC was directly coupled via nano electrospray to a Q Exactive Orbitrap mass spectrometer (Thermo Fisher Scientific). Capillary voltage was 2 kV. A top 12 method was used, the normalized fragmentation energy was 28%. Survey and fragment spectra were analyzed with Proteome Discoverer version 1.4 (Thermo Fisher Scientific) or Peaks Studio 7.5 (Bioinformatics Solutions, Waterloo, ON, Canada), respectively with the human sequence database from UniProt.

### Spot-test experiments

SC-galactose plates containing H_2_O_2_ (in a concentration range from 1–5 mM) and acetic acid (3–50 mM) were prepared to test for the sensitivity of yeast strains that are either overexpressing or are deleted for genes essential for the formation of LDs. These cells were then grown to stationary phase in liquid SC, were washed twice with water and serially diluted to OD_600_ values of 3.0; 1.0; 0.3; 0.1. 10 *μ*l aliquots were then spotted onto the plates prepared as described above. Sensitivity was determined by comparison of growth after 3 days at 28 °C.

### Isolation of mitochondria

HepG2 cells were grown to ~80% confluency in 75 cm^2^ cell culture flasks. Medium was aspirated and cells were washed 2 times with STE buffer (5 mM Tris pH7.4, 250 mM sucrose, 2 mM EGTA) before scraping them off an collecting them. Cells were resuspended in 5 ml of cold STE buffer and transferred to a 50 ml centrifuge tube. They were then centrifuged at 2000 r.p.m. for 10 min and 4 °C before removing the supernatant. Cells were resuspended in 2.5 ml cold STE buffer containing protease inhibitor and 0.5% BSA before transferring them to a potter homogenizer. They were then homogenized with 20–30 strokes and transferred to a 50 ml centrifuge tube. The homogenate was spinned down at 3000 r.p.m. for 3 min and 4 °C. The supernatant was then spinned down at 10 000 r.p.m. for 11 min at 4 °C, afterwards the supernatant was removed and the mitochondrial pellet was resuspended in ice cold STE before transferring it to a 15 ml centrifuge tube and topping it up with STE buffer. It was then spinned down at 11 600*×g* for 10 min and pellet was resuspended in 100 *μ*l of STE buffer.

### Nile red staining of yeast cells

Yeast strains were grown in YPD to mid-exponential phase and were then washed and resuspended in PBS. After exposure to heat shock (42 °C for 2, 4, 6, 8, 10, 12, 14 and 16 min) cells were fixed with 3.7% formaldehyde. In addition, yeast cells harboring either the plasmid pCM666-mBAX (murine BAX under control of a tet-inducible promoter) or pCM666 were incubated for 16 h in the presence of 10 *μ*g/ml doxycycline hyclate at 28 °C in SC-leu medium. All cells were then stained with 1 mg/ml Nile Red (Thermo Fisher Scientific, N-1142) and incubated in the dark for 20 min before measuring relative fluorescence with the multi-plate reader Anthos Zenyth 3100 (Anthos Labtec Instruments GmbH, Wals-Siezenheim, Austria).

### Generation of a quadruple mutant

Two deletion strains from the EUROSCARF library with different mating types were mated and selected on SD +his +leu +ura plates. Cells were then sporulated on SPO plates. The sporulated cells were incubated for 5 min at 37 °C with a 0.5 mg/ml zymolyase solution (Seikagaku Biobusiness, 20 T; Seikagaku Corporation, Tokyo, Japan). The spores were separated using a micromanipulator (Singer, MSM System series 200) on YPD plates. The strains carrying a double deletion were selected on YPD plates containing kanamycin. They were further analyzed by PCR with the respective primer pairs provided by EUROSCARF (Scientific Research and Development GmbH, Oberusel, Germany). The two resulting double mutants were then crossed with each other in exactly the same way resulting in a quadruple mutant strain.

### Statistical analysis

Data are reported as arithmetic means±S.E.M. Data were tested using unpaired two-tailed Student’s *t*-test, and results with *P*<0.05 were considered statistically significant.
