# Supplementary material for: Clearing the outer mitochondrial membrane from harmful proteins via lipid droplets
Source: Cell Death Discov. 2017 Mar 20;3:17016–. doi: 10.1038/cddiscovery.2017.16 (PMC5357670; doi:10.1038/cddiscovery.2017.16)
Supplement: Supplementary Information [file cddiscovery201716-s1.docx]

| Genotype | Genotype |
| --- | --- |
| BY4741; *MATa* pUG35-(V)BAX-GFP | BY4741; *MATa* p416GPD-MMI1-FLAG |
| BY4741; *MATa ERG6::RFP::KanMX4* | BY4741; *MATa* p416GPD-BAX-RFP pESC-HIS-ERG6-GFP |
| BY4741; *MATa LOA1::RFP::KanMX4* | BY4741; *MATa* pESC-HIS-MMI1-BAX |
| BY4741; *MATa ERG6::RFP::KanMX4* pUG35-(V)BAX-GFP | BY4741; *MATa* p416GPD-LRO1 |
| BY4741; *MATa LOA1::RFP::KanMX4* pUG35-(V)BAX-GFP | BY4741; *MATa* p416GPD-LRO1 YEplac181-MET25-(V)BAX-GFP |
| BY4741; *MATa* pUG35-(V)ERG6-GFP | BY4741; *MATa* p416GPD-LRO1 pESC-HIS-DGA1 |
| BY4741; *MATa* pUG35-(V)Bcl-XL-GFP | BY4741; *MATa* pCM666 |
| BY4741; *MATa* pCM666-mBAX | BY4741; *MATa* pCM666 p416GPD |
| BY4741; *MATa* pCM666-mBAX p416GPD | BY4741; *MATa* pCM666-mBAX p416GPD-LRO1 |
| BY4741; *MATa* pCM666 p416GPD-LRO1 | BY4741; *MATa* pESC-HIS |
| BY4741; *MATa* pESC-HIS-MMI1 | BY4741; *MATa* pESC-HIS-mBAX |
| BY4741; *MATa* pUG35-mBAX-GFP | BY4741; *MATa Δare1, Δare2, Δdga1, Δlro1* |
| BY4741; *MATa Δare1, Δare2, Δdga1, Δlro1* pYX142-mtRFP |  |

### Supplementary Table 1: Yeast strains created for this study
