# Supplementary material for: Clearing the outer mitochondrial membrane from harmful proteins via lipid droplets
Source: Cell Death Discov. 2017 Mar 20;3:17016–. doi: 10.1038/cddiscovery.2017.16 (PMC5357670; doi:10.1038/cddiscovery.2017.16)
Supplement: Supplementary Information [file cddiscovery201716-s2.docx]

| Vector | Primers | Enzymes | Method | Source | Destination Vector |
| --- | --- | --- | --- | --- | --- |
| pUG35 vMMI1 |  |  |  | (Rinnerthaler et al., 2013) |  |
| pUG35 vBAX | CCGGGATCCATGTTTGCTGATGGCAACTTC  CCATCGATCAGCCGCTCACGGAGG | BamHI; ClaI | PCR cloning | pCM666-mBAX | pUG35 |
| pUG35 mBAX | CTCTAGAATGGACGGGTCCGGGGAG  GGAATTCGCCCATCTTCTTCCAGAT | XbaI; EcoRI | PCR cloning | pCM666-mBAX | pUG35 |
| gen. ERG6 (kanMX-RFP) | AGCCAGAAAACGCCGAAACCCCCTCCCAAACTTCCCAAGAAGCAACTCAAGGAGCAGGGGCGGGTGC  CAATGAACGTGCTATCTTTTTATCTGCATATATAGGAAAATAGGTATATACCCCCTCGAGGTCGACGGTATCG |  | genomic integration | pRFP (Rinnerthaler et al., 2013) |  |
| gen. LOA1 (kanMX-RFP) | AGAGGAATTTTGTGAAGGAATATATCAGCGATCAACGTAAAAAGAGGAAGGGAGCAGGGGCGGGTGC  TGTTTGTTTCGATTAAATGCAATACATACTCCTAAAGACATATATATATACCCCCTCGAGGTCGACGGTATCG |  | genomic integration | pRFP (Rinnerthaler et al., 2013) |  |
| pUG35 vERG6 | CGGGATCCATGGGTGAGTGGAAG  GGAATTCTTGAGTTCCTTCTTGGGAAG | BamHi; EcoRI | PCR cloning | yeast genomic DNA | pUG35 |
| pEGFP-N3 vBAX | GGATTCATGTTTGCTGATGGCAACTTCAACTGG  GGGGTACCCAGCCGCTCACGGAGGAAGT | KpnI; EcoRI | PCR cloning | pCM666-mBAX | pEGFP-N3 |
| pIRES2 vBAX-GFP RFP | AACCATGTTTGCTGATGGCAACTTC  AAGGAAAAAAGCGGCCGCTTACTTGTACAGCTCGTCCA | NotI | PCR cloning | pUG35-vBAX | pIRES2 RFP |
| pIRES vBAX-GFP TOMM20-RFP | CTAGCGCTATGGTGGGCCGGAACAGCGC  GAAGATCTCCTTCCACATCATCCTCAGCCA | AfeI; BglII | PCR cloning | Rat cDNA | pIRES2 vBAX-GFP RFP |
| pIRES vBAX-GFP PLIN2-RFP | GCCGCCAGCGCTATGGCATCAGTAGCAGTGGATCCA  GCCGCCAGCGCTATCTGAGTTGTGA | AfeI | PCR cloning | Rat cDNA | pIRES2 vBAX-GFP RFP |
| pIRES vBAX-GFP PLIN3-RFP | CTAGCGCTATGTCTAGCAATGGGACAGAAGC  AGCGCTGCCTTCTCTTCAGGGGCTTTCT | AfeI | PCR cloning | Rat cDNA | pIRES2 vBAX-GFP RFP |
| pUG35 vBCL-XL | GGAAGCTTATGGGGGTAAACTGGGGTCGCATTGTG  GCGCGGAATTCGCTAGGTGGTCATTCAGGTAAGTGG | HindIII; EcoRI | PCR cloning | Rat cDNA | pUG35 |
| pEGFP-N3 vBCL-XL | GGAATTCATGCGGGATGGGGTAAATTGGGG  GGGGTACCGCCGCCGTCTTCCTGGATCC | KpnI; EcoRI | PCR cloning | Rat cDNA | pEGFP-N3 |
| pUG35 MMI1 |  |  |  | (Rinnerthaler et al., 2013) |  |
| p416GPD FLAG |  |  |  | (Mumberg et al., 1995) |  |
| pESC-HIS ERG6-RFP | CGGGATCCCGATGAGTGAAACAGAATTGAG  CCGCTCGAGCGGTTGAGTTGCTTCTTGGGAAGTTTG | BamHI; XhoI | PCR cloning | yeast genomic DNA | pESC-+HIS GFP |
| pESC-HIS GFP | CGACGCGTCGTTATTTGTACAATTCATCCA  CCGCTCGAGCGGATGTCTAAAGGTGAAGAATT | MluI; XhoI | PCR cloning | pUG35 | pESCC-HIS |
| pESC-HIS MMI1 (GAL1) | TATAGGGCCCGGGCGTCGACATGATTATTTACAAGGATATCTTCTC  GCGGTACCAAGCTTACTCGAGTTAGATCTTTTCTTCCACAATAC | SalI; HindIII | Gibson Assembly | yeast genomic DNA | pESC-HIS |
| pESC-HIS BAX (GAL10) | GGAATTCCATGGACGGGTCCGGGGAGCA  AAGGAAAAAAGCGGCCGCAAAAGGAAAATCAGCCCATCTTCTTCCAGA | EcoRI; NotI | PCR cloning | Rat cDNA | pESC-HIS |
| p416GPD LRO1 | CGGGATCCATGGGCACACTGTTTCGAAG  GGAATTCTTACATTGGGAAGGGCATCT | BamHI; EcoRI | PCR cloning | yeast genomic DNA | p416GPD |
| YEplac181 vBAX-GFP | CCCCCCGGGTTGGCCGATTCATTAATGCAGCTGGCAC  AACTGCAGATGACTCGACCAGTTATTTGTACAATTC | XmaI; PstI | PCR cloning | pUG35-vBAX | YEplac181 |
| YEplac181 |  |  |  | (Gietz and Sugino, 1988) |  |
| pESC-HIS DGA1 | CGCGTCGACTTACCCAACT  CGCGGATCCATGTCAGGAA | SalI; BamHI | PCR cloning | yeast genomic DNA | pESC-HIS |
| pIRES2 |  |  |  | Clontech |  |
| pIRES2 RFP | CCGCTCGAGATGACCATGATTACGCCAAGCG  ACGCGTCGACTTAGGCGCCGGTGGAGTGGC | XhoI; SalI | PCR cloning | pRFP | pIRES2 |
| pCM297 |  |  |  | (Rinnerthaler et al., 2012) |  |
| pCM297-mBAX | CCATCGATATATGGACGGGTCCGGGG  ATAAGAATGCGGCCGCTCAGCCCATCTTCTTC | NotI; ClaI | PCR cloning | Yep51-BAX | pCM297 |
| pCM666-mBAX | GAAGATCTAATTAGTGGAAGCTGAAAC  GAAGATCTCGTGTCGTTTCTATTATGAA | BglI | PCR cloning | yeast genomic DNA | pCM666 |
| p416GPD-RFP | GGAATTCATGACCATGATTACGCCAAG  CCCAAGCTTTTAGGCGCCGGTGGAGTGGC | EcoRI; HindIII | PCR cloning | pRFP | p416GPD |
| p416GPD-mBAX-RFP | CTCTAGAATGGACGGGTCCGGGGAG  GGAATTCGCCCATCTTCTTCCAGAT | XbaI; EcoRI | PCR cloning | pCM666-mBAX | p416GPD-RFP |
| pRFP |  |  |  | (Rinnerthaler et al., 2013) |  |
| Yep51 BAX |  |  |  | (Zha et al., 1996) |  |
| pUG35 |  |  |  | (Hegemann J.H., unpublished) |  |

### Supplementary Table 2: Vectors, Primers and Sources used for Cloning experiments
