# Supplementary material for: Clearing the outer mitochondrial membrane from harmful proteins via lipid droplets
Source: Cell Death Discov. 2017 Mar 20;3:17016–. doi: 10.1038/cddiscovery.2017.16 (PMC5357670; doi:10.1038/cddiscovery.2017.16)
Supplement: Supplementary Information [file cddiscovery201716-s3.docx]

Images of a primary hepatocyte containing the pIRES Plin3-RFP vBax-eGFP vector. (A) vBax-eGFP,(B) Plin3-RFP. Colocalisation of eGFP and RFP is indicated in white, differences in localisation in yellow arrows.
