# Supplementary material for: Clearing the outer mitochondrial membrane from harmful proteins via lipid droplets
Source: Cell Death Discov. 2017 Mar 20;3:17016–. doi: 10.1038/cddiscovery.2017.16 (PMC5357670; doi:10.1038/cddiscovery.2017.16)
Supplement: Supplementary Information [file cddiscovery201716-s4.docx]

Effect of staurosporine on LDs on HepG2 cells transfected with the vector pEGFP-N3-V-BAX. In (A) untreated cells and in (B) cells treted with 1 µM staurosporine are shown. A clear increase in LD numbers is obvious.
