# Supplementary material for: Clearing the outer mitochondrial membrane from harmful proteins via lipid droplets
Source: Cell Death Discov. 2017 Mar 20;3:17016–. doi: 10.1038/cddiscovery.2017.16 (PMC5357670; doi:10.1038/cddiscovery.2017.16)
Supplement: Supplementary Table 1 [file cddiscovery201716-s5.docx]

BY4741 cells transformed with vector Yeplac181-MET25-V-domain-BAX-GFP and yeast cells transformed with vector p416GPD-*LRO1* and vector Yeplac181-MET25-V-domain-BAX-GFP are shown in (A) and (B, respectively. A clear increase in LD numbers is obvious after overexpression of Lro1p (B).
