# Supplementary material for: Clearing the outer mitochondrial membrane from harmful proteins via lipid droplets
Source: Cell Death Discov. 2017 Mar 20;3:17016–. doi: 10.1038/cddiscovery.2017.16 (PMC5357670; doi:10.1038/cddiscovery.2017.16)
Supplement: Supplementary Table 2 [file cddiscovery201716-s6.docx]

BY4741 cells harboring LDs (upper line) and devoid of LDs (Δare1, Δare2, Δlro1,Δdga1) were spotted in different concentrations (OD600=3; OD600=1; OD600=0.3; OD600=0.1) onto plates containing 0 mM, 0.5 mM and 1 mM hydrogen peroxide. The strain devoid of LDs is sensitive to hydrogen peroxide.
