# Supplementary figures and images for: Clearing the outer mitochondrial membrane from harmful proteins via lipid droplets
Source: Cell Death Discov. 2017 Mar 20;3:17016–. doi: 10.1038/cddiscovery.2017.16 (PMC5357670; doi:10.1038/cddiscovery.2017.16)

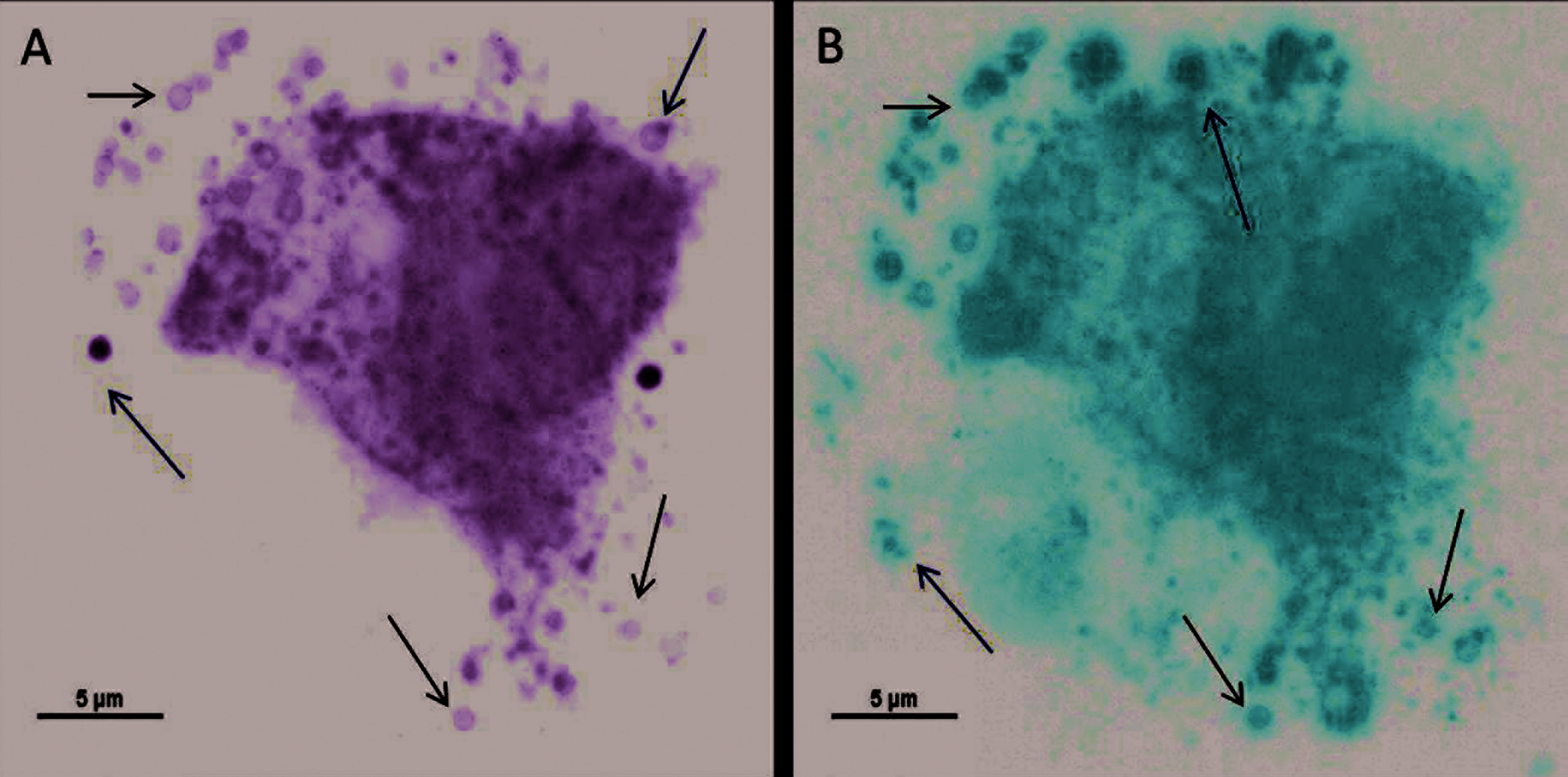

Supplement: Supplementary Figure 1 [file cddiscovery201716-s7.tiff]

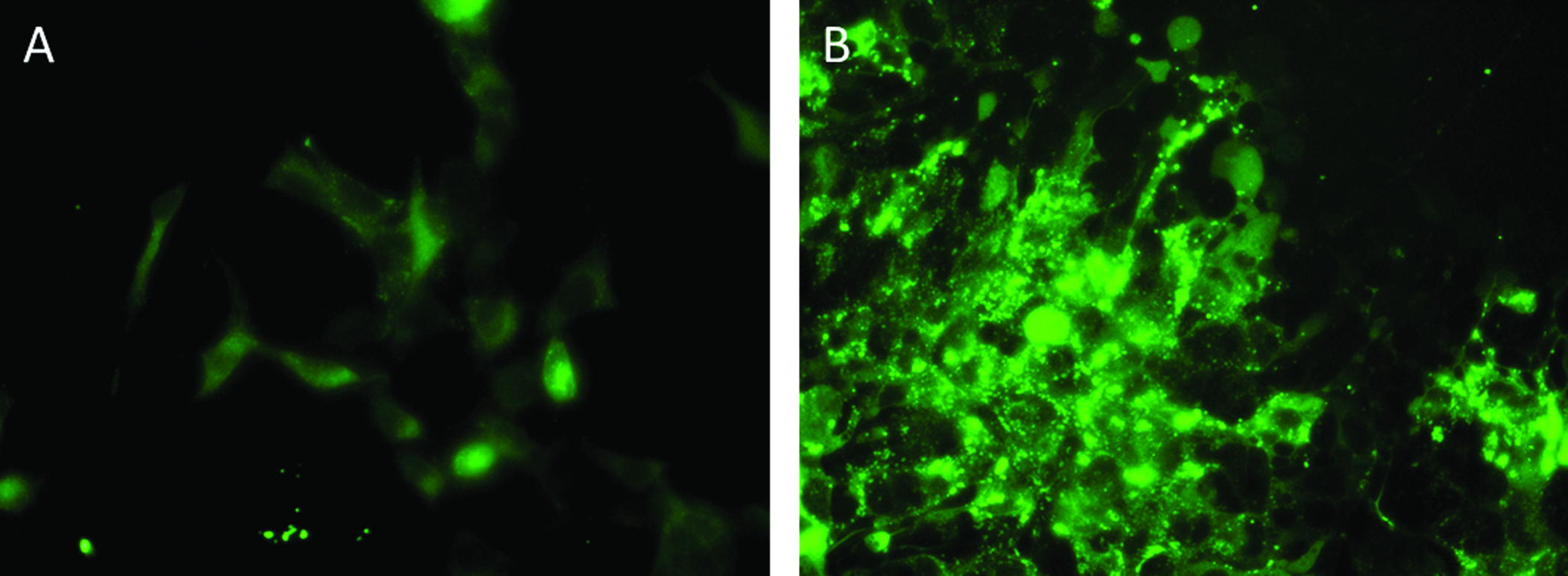

Supplement: Supplementary Figure 2 [file cddiscovery201716-s8.tiff]

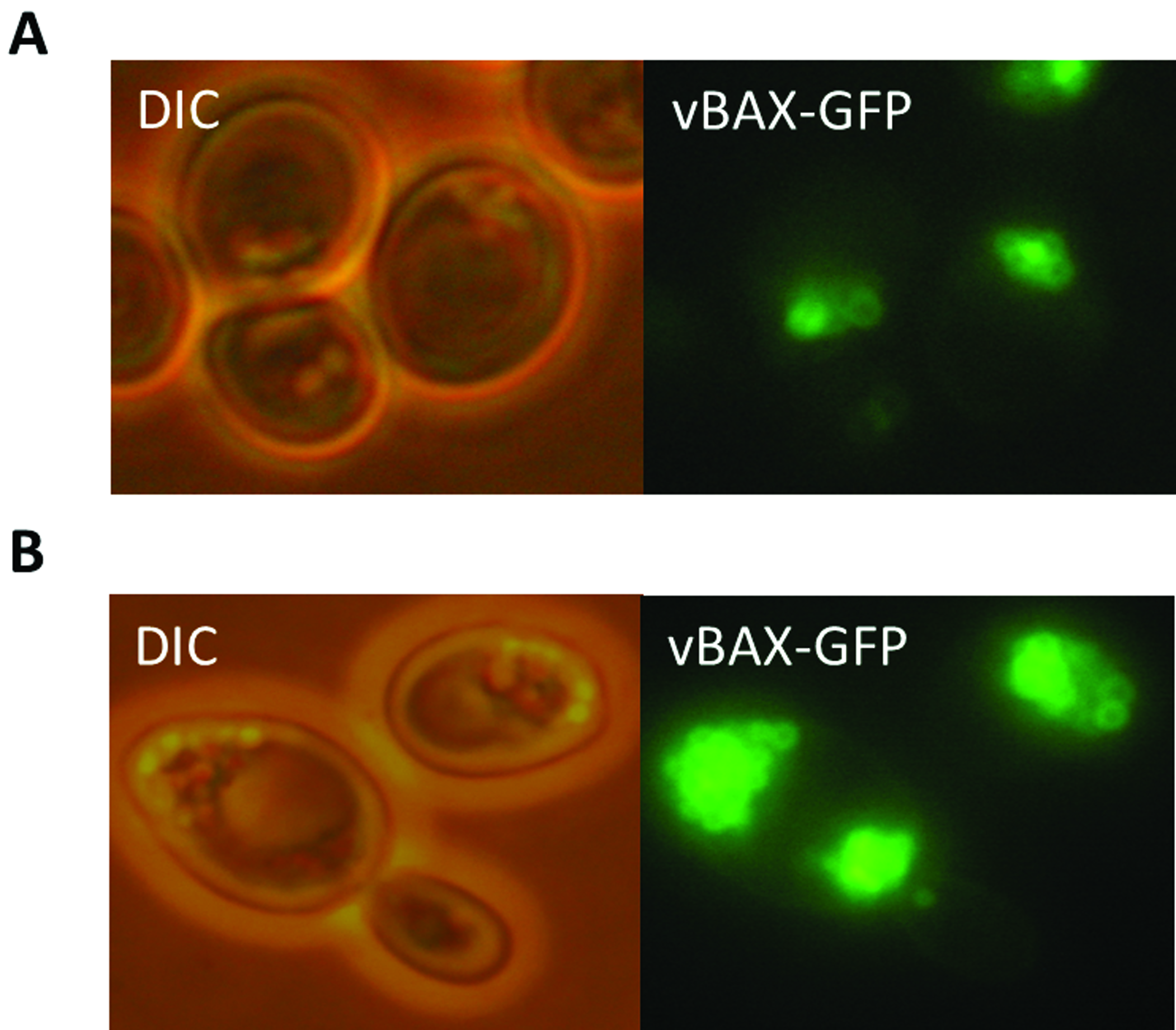

Supplement: Supplementary Figure 3 [file cddiscovery201716-s9.tiff]

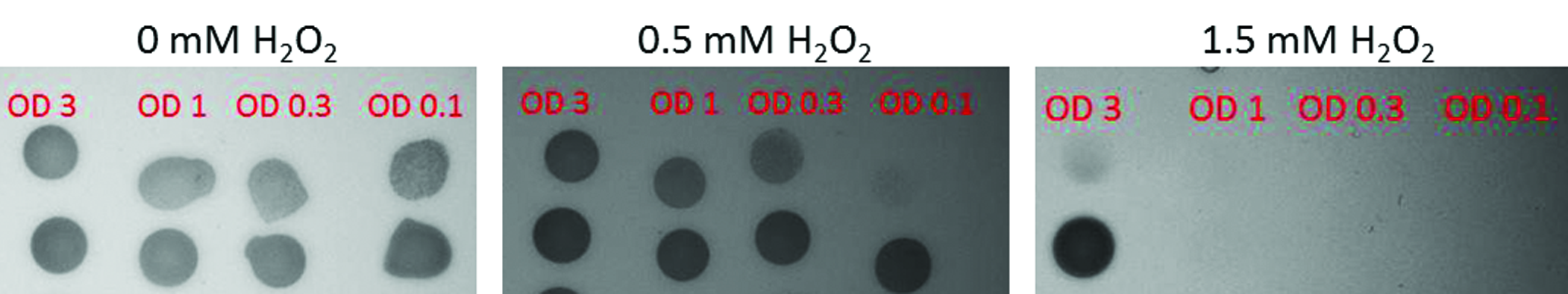

Supplement: Supplementary Figure 4 [file cddiscovery201716-s10.tiff]
